# Trends in the Aggressiveness of End-of-Life Care for Korean Pediatric Cancer Patients Who Died in 2007–2010

**DOI:** 10.1371/journal.pone.0099888

**Published:** 2014-06-12

**Authors:** June Dong Park, Hyoung Jin Kang, Young Ae Kim, MinKyoung Jo, Eun Sook Lee, Hee Young Shin, Young Ho Yun

**Affiliations:** 1 Department of Pediatrics, Seoul National University College of Medicine, Seoul, Korea; 2 Cancer Institute, Seoul National University College of Medicine, Seoul, Korea; 3 Hospital and Research Institute, National Cancer Center, Goyang, Korea; 4 Departmentof Biomedical Science, Seoul National University Hospital and College of Medicine, Seoul, Korea; National Cancer Institute, United States of America

## Abstract

**Background:**

In light of the Korean Supreme Court's 2009 ruling favoring a patient's right to die with dignity, we evaluated trends in aggressive care in a cohort of pediatric cancer patients.

Methods We conducted a population-based retrospective study that used administrative data for patients who died in 2007–2010 among the 5,203 pediatric cancer patients registered at the Korean Cancer Central Registry (KCCR) during 2007–2009.

**Results:**

In the time period covered, 696 patients died. The proportion who had received chemotherapy in the last 30 days of life decreased from 58.1% to 28.9% (*P*<0.001), those who received new chemotherapy in the same time period decreased from 55.2% to 15.1% (*P*<0.001), and those who received treatment in the last 2 weeks of life decreased from 51.4% to 21.7% (*P*<0.001). In the last 30 days of life, the proportion of patients whose hospital admission period was over 14 days increased from 70.5% to 82.5% (*P* = 0.03), the proportion who received cardiopulmonary resuscitation decreased from 28.6% to 9.6% (*P*<0.001), and we found no statistically significant trends in the proportion of emergency department visits, intensive care unit admissions, or mechanical ventilation.

**Conclusions:**

In this study, in contrast with earlier ones, the aggressiveness of end-of-life care of Korean pediatric cancer patients decreased dramatically.

## Introduction

Despite the dramatically improved outcomes for pediatric cancer patients following from significant advances in the disease's early detection and treatment over the past few decades [1], cancer is the leading cause of premature death in Korean children, accounting for 9%–11% of deaths from 2007 to 2010 [2]. As issues pertaining to end-of-life (EOL) care delivered to pediatric cancer patients affect a large number of family members[3]–[5], the quality of that care is of significant concern. In light of the Korean Supreme Court's 2009 ruling favoring a patient's right to die with dignity [6], avoidance of futile aggressive care of pediatric patients is essential, and support for families of children dying of cancer is crucial. However, knowledge about quality of care of children at EOL is poor [6], [7].

To better support children at EOL and their families, policies that provide appropriate care while making effective use of health care resources must be developed [7]. Earle and colleagues developed ways to use administrative data to monitor the quality of EOL care [8], and while some studies show national patterns [9]–[12], few evaluate the quality of EOL care that is delivered to children with cancer [7].

It is possible that the 2009 Korean Supreme Court ruling favoring a patient's right to die with dignity [6] may have led to a change in the aggressiveness of EOL care. As evaluating the quality of care of children at EOL is essential, we report the results of a population-based retrospective study that used administrative data to detect trends in aggressive care in a cohort of 696 pediatric cancer patients who died in South Korea from 2007 through 2010.

## Methods

### Study patients and database

Among 5, 203 pediatric cancer patients registered at the Korean Cancer Central Registry (KCCR) between 1 January 2007 and 31 December 2009, we analyzed the level of EOL care delivered to the 696 who died during that time. (The KCCR database covers 95% of newly diagnosed malignant tumors [13].)

As this study was sponsored by National Cancer Center Research Grant and we used national health data approved by National Cancer Center and National Health Insurance Cooperation that were not clinical data but secondary administration data such as data from the Korean Cancer Central Registry, National Statistical Office, and health insurance payment records from the Health Insurance Review & Assessment Service, we did not receive the approval from the ethics committee/institutional review board. However, we anonymized and de-identified patient records/information prior to analysis.

### Main outcome measures

We defined 1 month before death as 30 days, including the day of death, and 2 weeks before death as 14 days, including the day of death. We adopted the indicators of aggressive EOL care for terminal patients from Earle and colleagues [2], [8]. Main indicators were as follows: 1) starting a new chemotherapy regimen within the 30 days prior to death, 2) administering chemotherapy within the 30 days prior to death, 3) administering chemotherapy within the 14 days prior or death, 4) admitting to ER more than once within the 30 days prior to death, 5) hospitalizing more than 14 days within the 30 days prior to death, 6) admitting to hospital more than once within the 30 days prior to death, 7) intubating more than once during the 30 days prior to death [14], 8) mechanically intubating more than 14 days within the 30 days prior to death [14], 9) admitting to an intensive care unit (ICU) within the 30 days prior to death, and 10) performing cardiopulmonary resuscitation (CPR) within the 30 days prior to death [15].

Starting new chemotherapy was identified as the administration of a chemotherapeutic drug that had not been administered before. ER visits were identified by admission route code. Length of hospitalization counted admission and discharge on the same day as 1 day, and the frequency of admissions was obtained from medical bills. Incidences of intubation, mechanical ventilation, CPR, and ICU admission were identified from health insurance payment records and medical procedure codes. The level of use of medical services and progress variables were calculated as the mean per deceased patient. In addition, we collected demographic characteristics of patients (sex, age [0–1, 2–5, 6–10, or 11–17 years], residential area, type of cancer, cancer site status, and insurance).

### Statistical analysis

We used descriptive analysis to investigate the characteristics of the subjects and the distribution of the level of medical use and the Cochran-Armitage trend test to determine the statistical significance of yearly comparisons. We performed all statistical analyses with SAS v. 9.2 for Windows and considered *P*<0.05 as statistically significant.

## Results

Table 1 shows, by year of death, the characteristics of the 696 patients who met eligibility criteria. The statistically significant changes in aggressive care observed were a decrease in the use of chemotherapy within the last 14 and 30 days of life, a decrease in cardiopulmonary resuscitation within the last 30 days of life, and an increase in hospital admissions of >14 days during the last 30 days of life (Table 2).

**Table 1:** Characteristics of Patients by Year of Death (n = 696).

Characteristic	Year of Death							
	2007		2008		2009		2010	
	n	%	n	%	n	%	n	%
**Age, y**								
0–1	33	31.4	33	17.8	46	19.2	13	7.8
2–5	16	15.2	27	14.6	39	16.2	31	18.7
6–10	20	19.0	47	25.4	50	20.8	38	22.9
11–17	36	34.3	78	42.2	105	43.8	84	50.6
**Sex**								
Male	58	55.2	101	54.6	132	55	106	63.9
Female	47	44.8	84	45.4	108	45	60	36.1
**Place of residence**								
Metropolitan area	12	11.4	35	18.9	58	24.2	29	17.5
Small and medium city/Country	93	88.6	150	81.1	182	75.8	137	82.5
**Type of cancer**								
Leukemia	44	41.9	82	44.3	103	42.9	58	34.9
Brain tumor	20	19.0	47	25.4	64	26.7	41	24.7
Lymphoma	14	13.3	15	8.1	19	7.9	13	7.8
Others	27	25.7	41	22.2	54	22.5	54	32.5
**Site status**								
Localized only	24	22.9	46	24.9	59	24.6	42	25.3
Regional	6	5.7	13	7.0	10	4.2	12	7.2
Distant site(s)/node(s) involved	56	53.3	99	53.5	135	56.2	92	55.4
Unknown	19	18.1	27	14.6	36	15.0	20	12.0
**Insurance**								
National health insurance	85	81.0	149	80.5	225	93.8	154	92.8
Medical aid	20	19.0	36	19.5	15	6.2	12	7.2

**Table 2:** Trends in Indicators of Aggressive Care during the 4-Year Study Period (n = 696).

Aggressive care measure	n	% Patients receiving aggressive care measure					*P* ^1^
		Mean	2007	2008	2009	2010	
New chemotherapy regimen started in the last month of life	259	37.21	55.24	42.7	40.42	15.06	<0.01
Chemotherapy given in the last month of life	336	48.28	58.10	56.22	51.25	28.92	<0.01
Chemotherapy given in the last 2 weeks of life	292	41.95	51.43	49.19	46.25	21.69	<0.01
>1 ER visit in the last month of life	82	11.78	9.52	11.89	12.50	12.05	0.59
>14 days in hospital in the last month of life	539	77.44	70.48	76.76	77.50	82.53	0.03
>1 hospital admission in the last month of life	307	44.11	40.95	43.78	44.17	46.39	0.40
Admitted to the ICU in the last month of life	288	41.38	42.86	34.05	47.92	39.16	0.62
>1 intubation in the last month of life	61	8.76	14.29	7.03	9.17	6.63	0.12
Given CPR in the last month of life	113	16.24	28.57	14.05	17.08	9.64	<0.001
>14 days mechanical ventilation in the last month of life	111	15.95	12.38	15.68	18.33	15.06	0.50
^1^Cochran-Armitage trend test							

**Figure 1:**
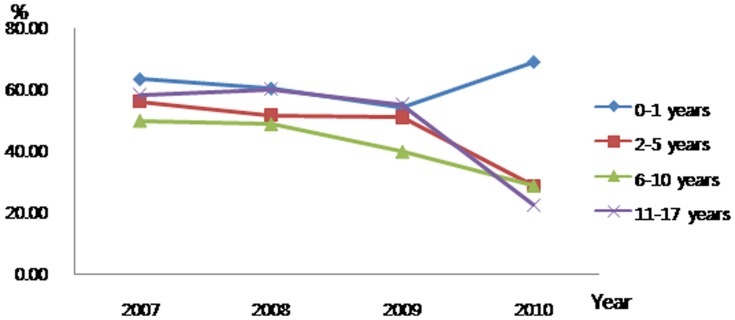
Trends for administering chemotherapy as aggressive end-of-life care to Korean pediatric cancer patients who died 2007–2010.

**Figure 2:**
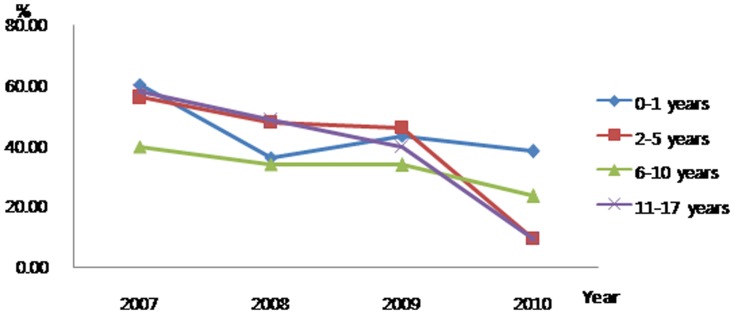
Trends for administering new chemotherapy as aggressive end-of-life care to Korean pediatric cancer patients who died 2007–2010.

**Figure 3:**
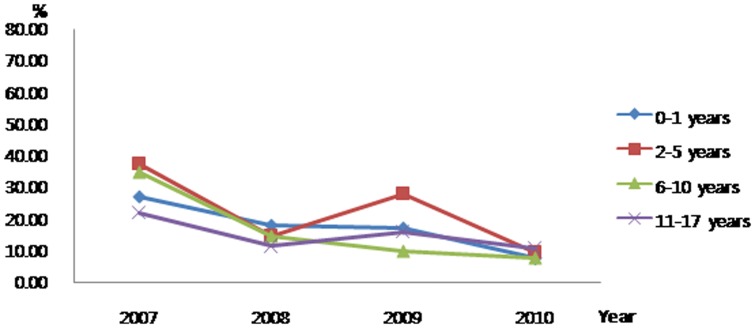
Trends for the administering CPR as aggressive end-of-life care to Korean pediatric cancer patients who died 2007–2010.

When examined by age group, significant findings were that the proportion of patients who received chemotherapy within the last 30 days of life decreased for 11–17-year-olds (Figure 1) while the proportion who received new chemotherapy in the same time period decreased for 2–5- and 11–17-year-olds (Figure 2). There seemed to be a trend to reduce CPR in the last 30 days of life (Figure. 3).

## Discussion

Our finding that about half the Korean pediatric patients with cancer who died 2007–2010 received chemotherapy in the last month of life is similar to earlier findings from Taiwan[7], [16]. A few studies about the rates of aggressive chemotherapy close to the end-of-life in the children with cancer have been reported [3], [4], [7], [17], [18].

The administration of new chemotherapy at EOL may reflect an attempt at salvage treatment. From the difference between the proportion of previously administered chemotherapy to new chemotherapy, we can speculate the rate of death due to chemotherapy-related complications. The increase of differences during study period suggests that the decreased rate of chemotherapy was mostly caused by no more introduction of salvage chemotherapy.

In Korea, decisions for the treatment of pediatric cancer patient are usually made by parents, who tend to pursue therapy even when they understand that there is no realistic chance for cure [19]. They want aggressive chemotherapy at any cost and even when major adverse effects are expected. That is in contrast to our findings in adult cancer patients, where 87% of family caregivers agreed to the withdrawal of futile life-sustaining treatments and 70% supported withholding of them [20]. The proportion of pediatric cancer patients who received life-sustaining treatment at EOL in Korea was comparable with that of the United States [5], [7], [17] and Taiwan [5], [7], [17]. The rate of CPR attempts in pediatric terminal cancer patients was about 7%–7.6% in the United States [5], [21], [22] and 25% in Japan [5], [21], [22]. We believe that the high rate of EOL ICU care in Korea is based on culture and that the relatively high rate of patients who received mechanical ventilation during their last month of life was due to the complication of chemotherapy itself; those rates did not change during the study period.

The decrease of aggressiveness in treating pediatric cancer patients at EOL may have been due to changes of attitude toward EOL care [20]. That decrease was observed before 2009, when the Korean Supreme Court ruled in favor of a patient's right to die with dignity. Since the ruling, however, there was a dramatic decrease in treatments given during the last month of life, including new chemotherapy regimens started and chemotherapy administered. The decrease in futile treatments might also be related to a 2009 change in national medical insurance coverage, which thereafter paid only for cancer treatments that were evidence-based. We do believe that medical insurance policy can influence population attitudes to EOL care.

The increasingly high proportion of study patients who were admitted to a hospital for more than 14 days during their last 30 days of life may have been due to Korea's changing social environment. With the current trend being toward two-generation families and more women holding jobs, having a dying child at home can be difficult, but Korea does not have sufficient hospice care facilities for children. And we believe that the increase in the proportion of patients who were admitted to a hospital within the last 30 days of life in the 0–1-year-old age group was due to the effect of complications of intensive chemotherapy in that age group.

A limitation of our study is that there are no data about hospice care, but hospice care in Korea is rare and not covered by national medical insurance. Indeed, the lack of hospice care may explain why EOL patients are admitted to hospitals in Korea. Another limitation is that the study covers only patients who died in a hospital—the only data available through our insurance system—and the data for aggressive EOL care administered outside of a hospital may differ. In this study we couldn't review the medical records of patients directly, so it was nearly impossible to find out exact causes of death. Finally, we did not analyze the whole scenario starting with the time of diagnosis and early treatment. Therefore, this study did not reflect overall quality of care the whole scenario from the beginning

The strength of our study lies in its population-based sample, which encompassed all types of cancer in Korean children of all ages. Future studies should focus on interventions that can influence parents' attitudes on aggressive EOL care of pediatric cancer patients, and on family-centered planning.
